# Subthreshold resonance in biophysically-based models of low- and high-input conductance motoneurons

**DOI:** 10.1186/1471-2202-16-S1-P129

**Published:** 2015-12-18

**Authors:** Vitor M Chaud, André F Kohn

**Affiliations:** 1 Biomedical Engineering Laboratory, Dept. of Telecommunication and Control Engineering, University of São Paulo, São Paulo, SP, 05508-900, Brazil

## 

Resonance in the membrane potential enables a neuron to discriminate its inputs on the basis of their frequency content, so that oscillatory inputs near the resonant frequency produce the largest responses [1]. Recently, Manuel et al. [2] have shown that spinal motoneurons (MNs) exhibit membrane resonance in the frequency range of about 8-14 Hz. Modeling studies of resonance in neural membrane usually use minimal or reduced models, accounting only for the ionic currents sufficient to generate the resonance and plausible to exist in the investigated neuron type. However, in the present study we developed models of low- and a high-input conductance MNs including most of the ionic currents known or hypothesized to exist in mammalian MNs and exhibiting several of the known physiological features. Therefore, instead of investigating resonance as an isolated phenomenon we analyze it as consequence of interactions between the passive properties and the ionic currents in a more realistic fashion. The low-input conductance MN model did not show resonances, whereas the high-input conductance MN model showed resonances dependent on the membrane potential (Figure 1). These resonances were mainly caused by the hyperpolarization-activated cationic (H) current. However, for membrane potentials near the firing threshold (purple lines in Figure 1), another resonance mainly affected by transient sodium and delayed rectifier currents predominated and its resonance frequency was correlated with the minimum firing rate caused by a step current injection.

**Figure 1:**
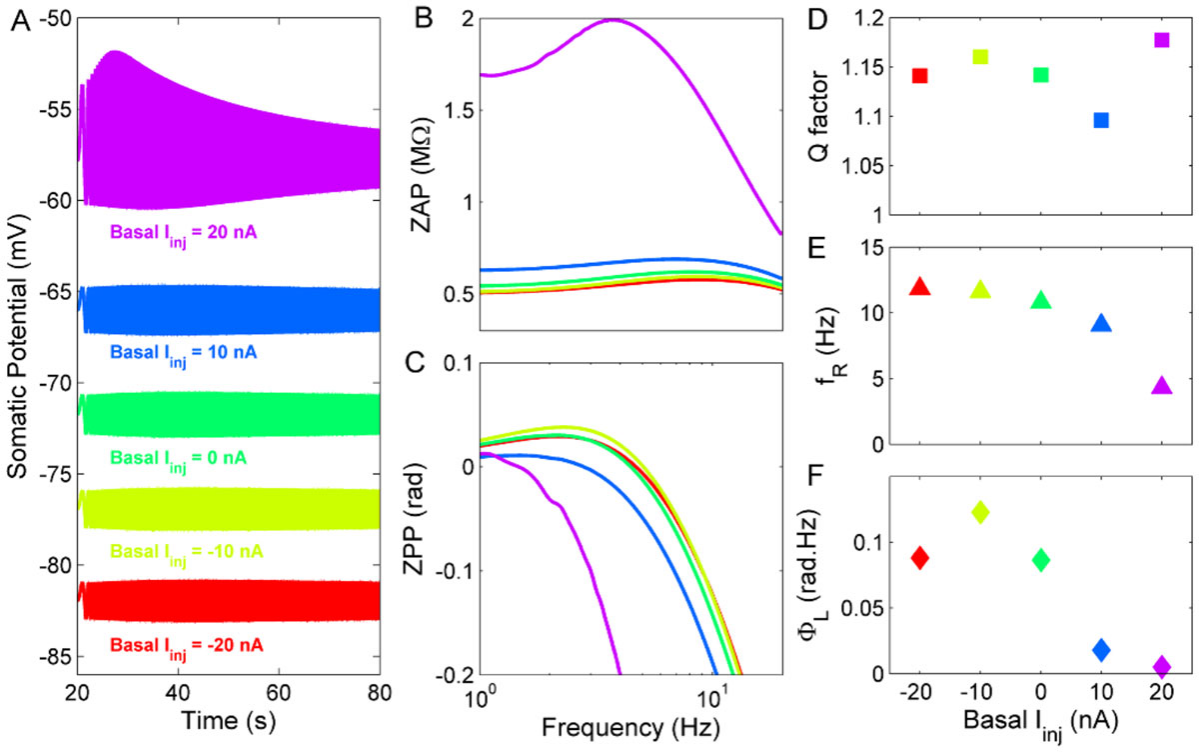
**Dependence of resonance of the high-input conductance MN model on the basal level of injected current**. **A**. somatic potential **B**. impedance amplitude **C**. impedance phase **D**. Quality (Q) factor **E**. resonance frequency (f_r_) **F**. total phase advance (Φ_L_).

## Conclusion

These results contribute to the understanding of the complex interactions between active and passive properties in MNs and how these interactions can produce membrane potential resonance and affect action potential generation. The relation we found between subthreshold resonance and the minimum firing rate merits further studies to better understand how firing rate may be affected by membrane resonances.
